# Detoxification activity of bioactive food compounds against ethanol‐induced injuries and hangover symptoms: A review

**DOI:** 10.1002/fsn3.3520

**Published:** 2023-06-30

**Authors:** Masoumeh Moslemi, Behrooz Jannat, Maryam Mahmoudzadeh, Mehran Ghasemlou, Abdol‐Samad Abedi

**Affiliations:** ^1^ Halal Research Center of IRI Ministry of Health and Medical Education Tehran Iran; ^2^ Nutrition Research Center and Department of Food Science and Technology, Faculty of Nutrition and Food Science Tabriz University of Medical Sciences Tabriz Iran; ^3^ School of Science STEM College, RMIT University Melbourne Victoria Australia; ^4^ Department of Research Deputy, National Nutrition and Food Technology Research Institute, Faculty of Nutrition Sciences and Food Technology Shahid Beheshti University of Medical Sciences Tehran Iran

**Keywords:** anti‐hangover agents, bioactive food compounds, detoxification, ethanol, food supplementation, nutrient deficiency

## Abstract

Alcohol drinking is a popular activity among adolescents in many countries, largely due to its pleasant, relaxing effects. As a major concern, ethanol consumption put the drinkers at risk of nutrients' deficiency due to the disordered eating, anorexia, and malabsorption of nutrients. Moreover, alcohol drinking may lead to the development of hangover symptoms including diarrhea, thirsty, fatigue, and oxidative stress. A broad range of functional food components with antioxidant and/or anti‐inflammatory properties including pectin, aloe vera polysaccharides, chito‐oligosaccharides, and other herbal components have been explored due to their detoxification effects against ethanol. The underlying anti‐hangover mechanisms include reducing the intestinal absorption of ethanol or its metabolites, increasing the activity of ethanol metabolizing enzymes, development of fatty acid β‐oxidation in mitochondria, inhibition of inflammatory response, blocking the target receptors of ethanol in the body, and possession of antioxidant activity under the oxidative stress developed by ethanol consumption. Therefore, the development of bioactive food‐based therapeutic formula can assist clinicians and also drinkers in the alleviation of alcohol side effects.

## Graphical abstract

A broad range of functional food components with antioxidant and/or anti‐inflammatory properties have been explored that can induce detoxification effects against ethanol. The underlying mechanisms of these bioactive food components include reducing the intestinal absorption of ethanol or its metabolites, increasing the activity of ethanol metabolizing enzymes, and possession of antioxidant activity under the oxidative stress developed by ethanol consumption.

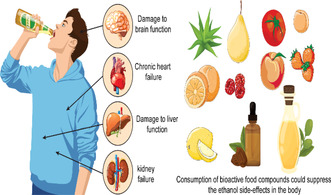

## 1 INTRODUCTION

Alcohol drinking is a major public health issue in most societies. According to the World Health Organization (WHO), about 3 million deaths worldwide and 5.1% of the global burden of diseases are caused by alcohol abuse every year (World Health Organization, 2018). The communication of the risks associated with alcohol use or misuse is becoming more important, particularly in the era of the COVID‐19 outbreak. Several studies have highlighted the health benefits of low‐to‐moderate drinking in reducing the risks of cardiovascular disease (Di Castelnuovo et al., 2017; Fernando, 2017; Minzer et al., 2020; Shah et al., 2018). However, other studies suggest that even low‐to‐moderate alcohol intake might still pose some risk (Charlet & Heinz, 2017; O'Keefe et al., 2018; Wilson & Braillon, 2018). Despite the varying results of numerous studies, owing to the alcohol's harmful and long‐lasting physical, mental, chemical, and behavioral implications, which cause a substantial financial burden on health‐care systems, most governments have deemed alcohol unsafe and taken steps to ensure that convenient access to alcohol is limited in line with the WHO action plan 2013–2020 (Burton & Sheron, 2018; Moslemi et al., 2020; Rekve et al., 2019).

The scientific literature indicates that alcohol metabolism can have a significant effect on the absorption of essential nutrients. Alcohol, once ingested into the body, is mainly absorbed in the upper intestinal tract by diffusion, promoting gastrointestinal inflammation (Kamran et al., 2020; Thomson et al., 2017). The alcohol‐induced intestinal inflammation may be a potential contributor to organ failure, such as chronic liver disease, or immune dysfunctions. Acute alcohol ingestion is often associated with the development of combined physical and mental symptoms such as dizziness, headache, fatigue, and muscle pain; these symptoms are known as hangover effects (Sæther et al., 2019; Verster et al., 2010).

**FIGURE 1:**
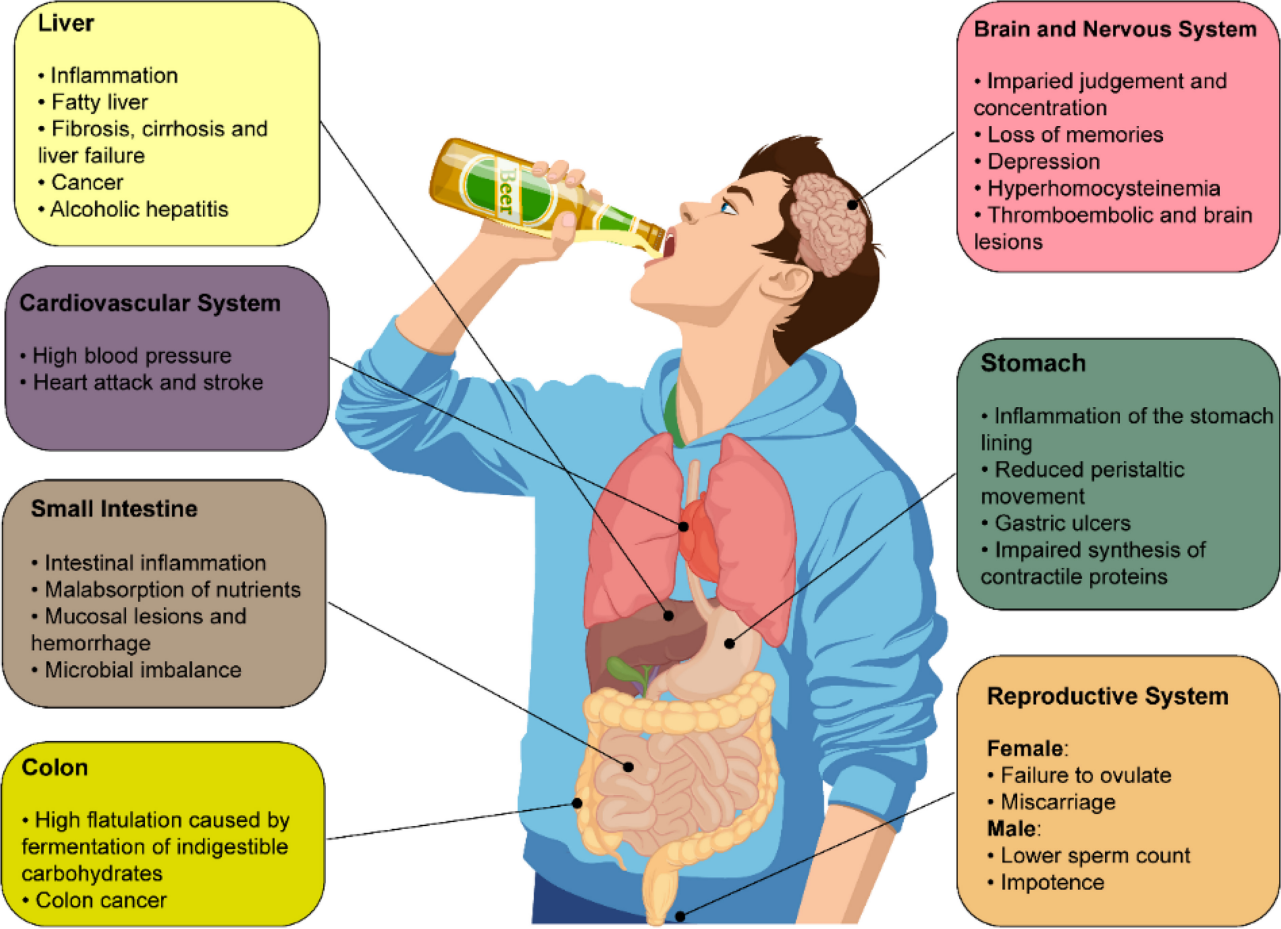
A summary of potential long‐term health effects of excessive alcohol drinking on some main organs.

Alcohol metabolism appears to depend on a number of criteria such as race/ethnicity, sex, age, disease status, nutritional status, amount of the consumed alcohol, and duration of alcohol exposure (Cederbaum, 2012; Rajendram & Preedy, 2005). Given the high reactivity of ethanol to agents in the environment, it can be easily absorbed, mainly in the upper intestinal tract, and then is distributed to different organs, where it can impair several biological processes (Figure 1).

The home‐based detoxification of drinkers with hangover symptoms without a need for hospitalization is critical to prevent organ damage or unintended death. Several studies have shown that natural food components and medicinal extracts with antioxidant properties can relieve the severe symptoms of alcohol hangovers (Cui et al., 2014; Czech & Seki, 2020; Srinivasan et al., 2019; Uto‐Kondo et al., 2018, 2020). The underlying detoxification mechanisms of natural food extracts in suppressing hangover symptoms are not well documented. In response to this gap in the literature, this paper aims to critically overview a broad range of food supplements and functional food components with antioxidant and/or anti‐inflammatory properties that can alleviate ethanol disorders and induce ethanol detoxification effects.

## 2 PATHWAYS OF ETHANOL METABOLISM

**FIGURE 2:**
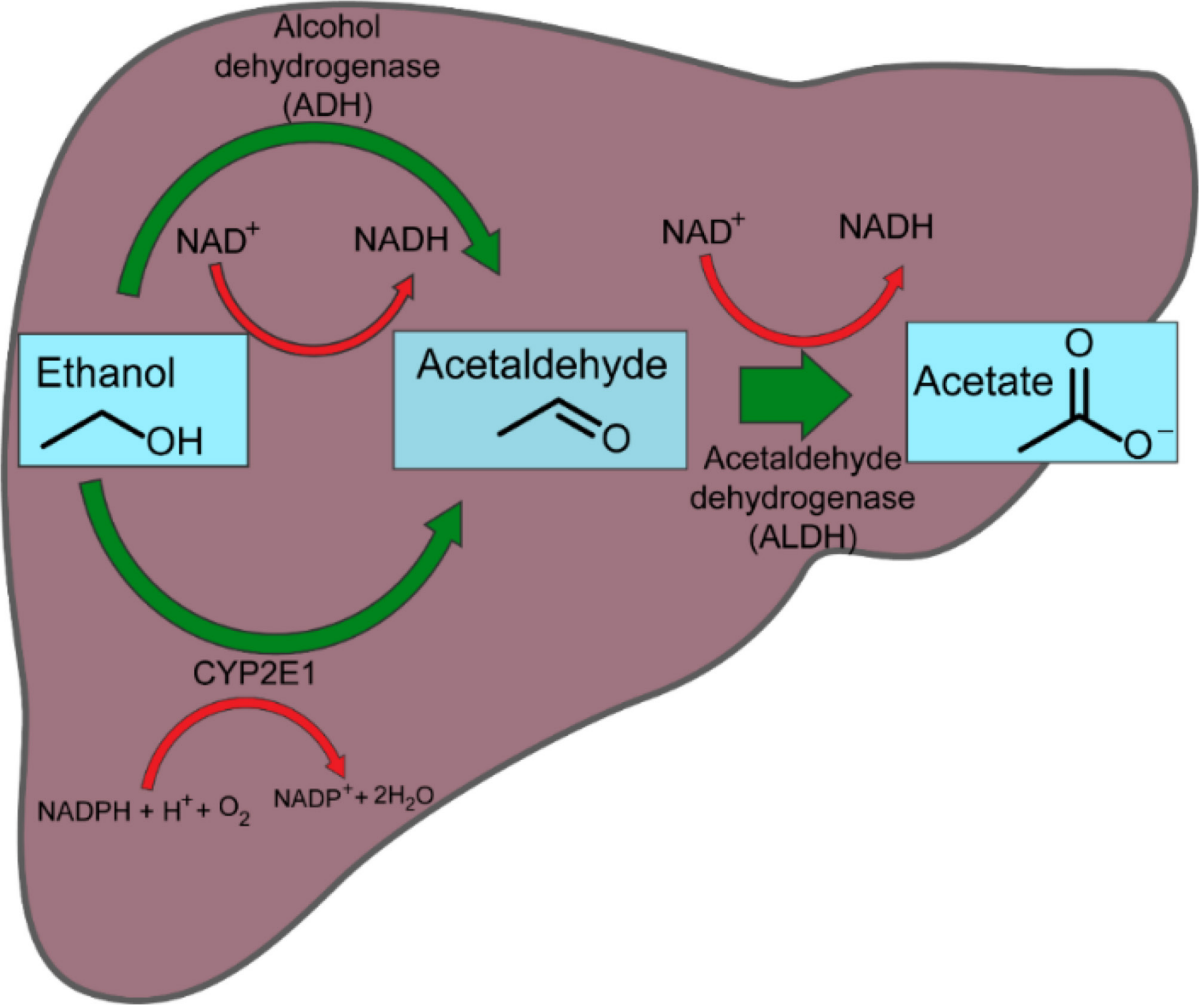
Metabolism of ethanol into acetaldehyde by two enzymatic pathways. In the liver, alcohol dehydrogenase (ADH) plays a major role, while cytochrome P450 (CYP2E1) plays a minor role.

In humans, ingested alcohol is primarily absorbed from the stomach and the small intestine. The liver is the major organ responsible for metabolizing up to 90% of ingested alcohol. There are two main pathways that enable metabolization of ethanol: alcohol dehydrogenase (ADH) and cytochrome P4502E1 (CYP2E1). ADH catalyzes the oxidation of ethanol to acetaldehyde, which is further oxidized to acetate by aldehyde dehydrogenase (ALDH) (Figure 2). In addition, catalase oxidation in the third pathway especially in the brain and when oxidative stress develops (Jiang et al., 2020; Wilson & Matschinsky, 2020).

ADH and ALDH, two NAD^+^‐dependent enzymes, use the conversion of the cofactor nicotinamide adenine dinucleotide (NAD^+^) to its reduced form (NADH). In the first reaction, ethanol loses a proton (H^+^), and a hydride ion (H^−^) is transferred from the ethanol to NAD^+^ to reduce it to NADH, resulting in an enhanced NADH/NAD^+^ ratio. During excessive alcohol consumption, CYP2E1 accompanies ADH in ethanol metabolism. In this catalytic cycle, a significant amount of reactive oxygen species (ROS) is produced, inducing an oxidative stress (Cederbaum, 2010; Guengerich & Avadhani, 2018; Kawaratani et al., 2013; Mohn & Johnson, 2015). Most of these oxidative pathways occur in the liver. Thus, the liver is the organ most vulnerable to alcoholism (Haynuk & Sheremeta, 2019).

## 3 ALLEVIATION OF ETHANOL‐INDUCED INJURIES BY FOOD SUPPLEMENTATION

**FIGURE 3:**
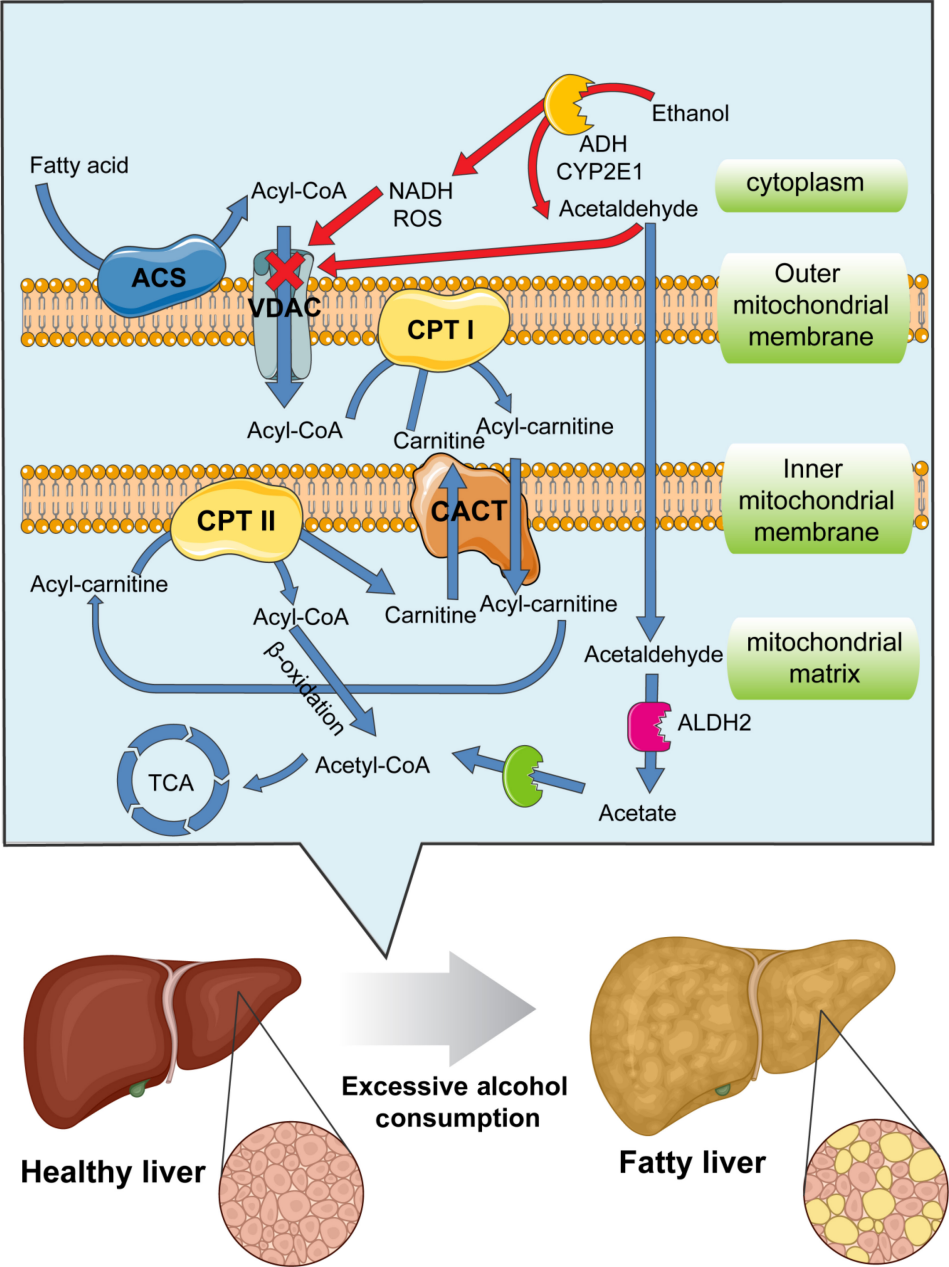
Mitochondrial fatty acid beta‐oxidation and its role in ethanol‐induced fatty liver. Fatty acids enter into the cell. In the cytoplasm, fatty acids are converted to fatty Acyl‐CoA by acyl‐CoA synthase and then transferred to the mitochondrial matrix by VDAC, CPT I, CACT, and CPT II, step by step. Acyl‐CoA then undergoes β‐oxidation to produce acetyl‐CoA for tricarboxylic acid cycle. These are the normal mechanisms for fatty acid catabolism. In the presence of alcohol, these mechanisms are impaired, resulting in morphologic changes in the liver cells and the accumulation of fats. ACS, Acyl‐CoA synthase; CPT, carnitine O‐palmitoyltransferase; CACT, carnitine‐acylcarnitine translocase; VDAC, voltage‐dependent anion channel.

Increasing evidence suggested that excessive alcohol consumption could affect the availability of nutrients, particularly when alcohol‐induced vomiting, degenerated intestinal epithelium, anorexia, or diarrhea occur (Bode & Bode, 2003; Seitz & Suter, 2002). Research has identified a number of possible explanations for the co‐occurrence of alcohol use and disordered eating. Alcohol, once consumed, suppresses the appetite in the alcoholics by elevating the secretion of tumor necrosis factor‐α (TNF‐α) and leptin (the satiety hormone secreted by adipose tissues). This is followed by upregulation of the secondary inflammatory factors including interleukin‐1β (IL‐1β), interleukin‐6 (IL‐6), and interleukin‐8 (IL‐8) by TNF‐α, which further suppresses appetite (Kamran et al., 2020). Followed by diarrhea induced by ethanol, the lower sodium concertation leads to reduced activity of sodium–potassium ATPase and glutathione/glucose cotransporters on the intestinal cell surface (Butts et al., 2019). Moreover, excessive alcohol prevents protein synthesis in the small intestine: Contractile proteins are less likely to be synthesized in the gastrointestinal (GI) tract of alcohol drinkers in which peristaltic movements and transition time of food are adversely affected (Thomson et al., 2017). These malnutrition and malabsorption are exacerbated in the drinkers when metabolism of essential nutrients is affected. For example, folate is an important vitamin for the development of fetal nervous system and its absorption is largely affected by alcohol consumption. Persson et al. (2013) reported that high folate intake could be helpful in ameliorating alcohol side effects and preventing alcohol‐associated liver diseases. Folate deficiency can lead to acute ethanol‐induced diarrhea because it facilitates an increase in cyclic adenosine monophosphate concentrations by stimulating and activating adenylate cyclase in the intestinal mucosa (Bode & Bode, 2003). Carnitine plays an essential role in the transfer of fatty acids into the mitochondria for their metabolism (Figure 3). Considering the high prevalence of malnutrition and malabsorption in heavy drinkers of alcohol, their bodies often suffer from the depletion of carnitine and other micronutrients such as lysine and methionine (precursors of carnitine). Given the crucial role of carnitine in facilitating fatty acid metabolism, its reduced levels can significantly affect hepatic fatty acid beta‐oxidation in alcoholics. Therefore, carnitine supplementation or consumption of animal protein may be helpful in prevention of ethanol‐induced liver damages (Wilson & Matschinsky, 2020).

Vitamin D deficiency due to less exposure of the drinkers to sunlight and disordered enzymatic metabolism of the vitamin (Gonzalez‐Reimers et al., 2017), calcium deficiency as a result of vitamin D depletion (Rajendram & Preedy, 2005), B‐group vitamin deficiency due to their malabsorption (Miyazaki et al., 2012; Rajendram & Preedy, 2005; Seitz & Mueller, 2014; Subramanya et al., 2010), and vitamin A and vitamin E deficiencies in response to the oxidative stress after alcohol intake (Gonzalez‐Reimers et al., 2014) are of other nutritional problems after alcoholism which can be ameliorated by food supplementation.

## 4 ALLEVIATION OF ETHANOL‐INDUCED INJURIES AND HANGOVER SYMPTOMS BY BIOACTIVE FOOD COMPOUNDS

Alcohol hangover refers to the combination of mental and physical symptoms when increased alcohol metabolism and formation of metabolites in the body lead to reduced blood alcohol concentrations. The hangover symptoms may often last for 24 h after alcohol consumption (Verster et al., 2010). Several hangover treatments have been proposed to reduce the sudden rise in inflammatory mediators' concentrations after heavy drinking (Khan et al., 2019; Shati & Elsaid, 2009; Yan et al., 2014). If alcohol is consumed along with the food, it contributes to the formation of free radicals and the activation of pro‐carcinogens by induction of CYP2E1. Ethanol can serve as a solvent to dissolve the carcinogens that are considered a contributing risk factor in promoting esophageal cancer and facilitating their penetration into the aerodigestive mucosa (Alzeer & Abou Hadeed, 2016; Husain et al., 2011). Acetaldehyde and other generated aldehydes during oxidative stress can cross‐react to form hybrid adducts composed of different combinations of acetaldehyde‐protein or acetaldehyde‐DNA in the tissues. The clinical significance of this phenomenon is accelerated when alcohol is consumed with high levels of fat (which is susceptible to oxidative conditions) or iron‐enriched food (which acts as a pro‐oxidant) (Hyun et al., 2021; Niemela, 2001). However, one potential treatment can be the use of dietary bioactive agents to eliminate or minimize the development of next‐day hangover symptoms. These bioactive compounds facilitate alcohol detoxification by stimulating the activity of the enzymes involved in alcohol metabolism, serving as strong antioxidants to suppress toxicity and ethanol‐induced oxidative stress, and reduce the chance of alcohol uptake in the GI tract (Lu et al., 2020; Ozkol et al., 2017; Srinivasan et al., 2019). As reported, the hangover symptoms arisen from alcohol consumption are mainly driven by acetaldehyde. Importantly, the bioactive components could be specifically served as functional ingredients exhibiting positive effects on ALDH activity, thereby promoting more conversion of acetaldehyde to acetic acid. Indeed, this can greatly help in the immediate clearance of acetaldehyde and ethanol from the body (Srinivasan et al., 2019). In the next section, we review the scientific mechanisms of some selected food components that have exhibited significant anti‐hangover effects through detoxification of alcohol metabolites (Table 1).

**TABLE 1:** A summary of anti‐hangover food compounds and the underlying mechanisms for alleviation of ethanol side effects in the drinkers.

Food compound	Anti‐hangover mechanism	References
Pectin	High affinity to ethanol, which makes it unavailable for metabolizing enzymes Low absorption of ethanol due to the viscosifying effect of pectin	Sheremeta & Haynuk (2018)
Aloe vera polysaccharides	Increasing the antioxidant potential Inhibition of inflammatory response Development of fatty acid β‐oxidation	Cui et al. (2014)
Chito‐oligosaccharides	Increased activity of ALDH Development of fatty acid β‐oxidation Increasing the activity of glutathione peroxidase	Bailey et al. (2001), Cho et al. (2010), Videla & Guerri (2017)
Herbal medicines (*Camellia sinensis, Houttuynia cordata*, *Nelumbo nucifera* G., *Viscum album* L., *Lycium chinense* L., *Inonotus obliquus*, *Acanthopanax senticosus* H., *Glycyrrhiza uralensis*, *Pueraria thunbergiana*, *Liriope platyphylla*, *Polypori umbellati*, *Ixeris dentata*, *Acanthopanax sessiliflorus*, *Artemisia capillaris* Thunb, *Paeonia lactiflora* Pallas, *Plantago asiatica* L., *Malpighia glabra*, *Opuntia ficus indica*, *Ginkgo biloba*, *Salix alba*, *Zingiber officinale*)	Activation of ADH and ALDH Inhibition of pro‐inflammatory mediators Development of antioxidant activity	Lee, Kim, et al. (2009), Lieb & Schmitt (2020), Noh et al. (2009), Yoon et al. (2011), You et al. (2016)
Minerals	Acting as cofactor of antioxidant enzymes Multivalent metals take part in oxidation–reduction reactions Blocking the target receptors of ethanol in the brain	Min et al. (2010), Ron & Wang (2009), Smith et al. (2009)
Ginseng	Suppression of ethanol‐induced oxidative stress by the antioxidant compounds	Lee et al. (2014), Park et al. (2019), Sharma et al. (2020)
*Hovenia dulcis* tree	Increasing the activity of ADH and ALDH Development of antioxidant potential Anti‐nitration effect of flavonoid compounds in favor of improved metabolism of ethanol by catalase Suppression of pro‐inflammatory agents and increased level of anti‐inflammatory compounds Blocking the target receptors of ethanol	Je et al. (2021), Kang et al. (2021), Kim et al. (2017), Wang et al. (2016)
Fermented persimmon juice	Role of GABA in the increased activity of ADH and ALDH, decreasing the ethanol absorption, and suppression of oxidative stress	Boby et al. (2021); Cha et al. (2011); Zhou et al. (2019); Zhu et al. (2019)
Shiitake extract	Reducing the absorption of ethanol and acetaldehyde in the GI tract by slowing the peristaltic movement	Uto‐Kondo et al. (2020)

### 4.1 Pectin

Pectin is a multifunctional and non‐digestive biopolymer, popularly known as dietary fiber, which serves a wide range of roles including antimicrobial activities, carrier for nutraceutical and drug delivery, and binders in biological systems (Cheraghali et al., 2018; Moslemi et al., 2014, 2018). The most attractive property of pectin lies in its manifold affinity to ethanol, which allows it to neutralize and detoxify ethanol's metabolites and alleviate ethanol‐induced disorders (Sheremeta & Haynuk, 2018).

**FIGURE 4:**
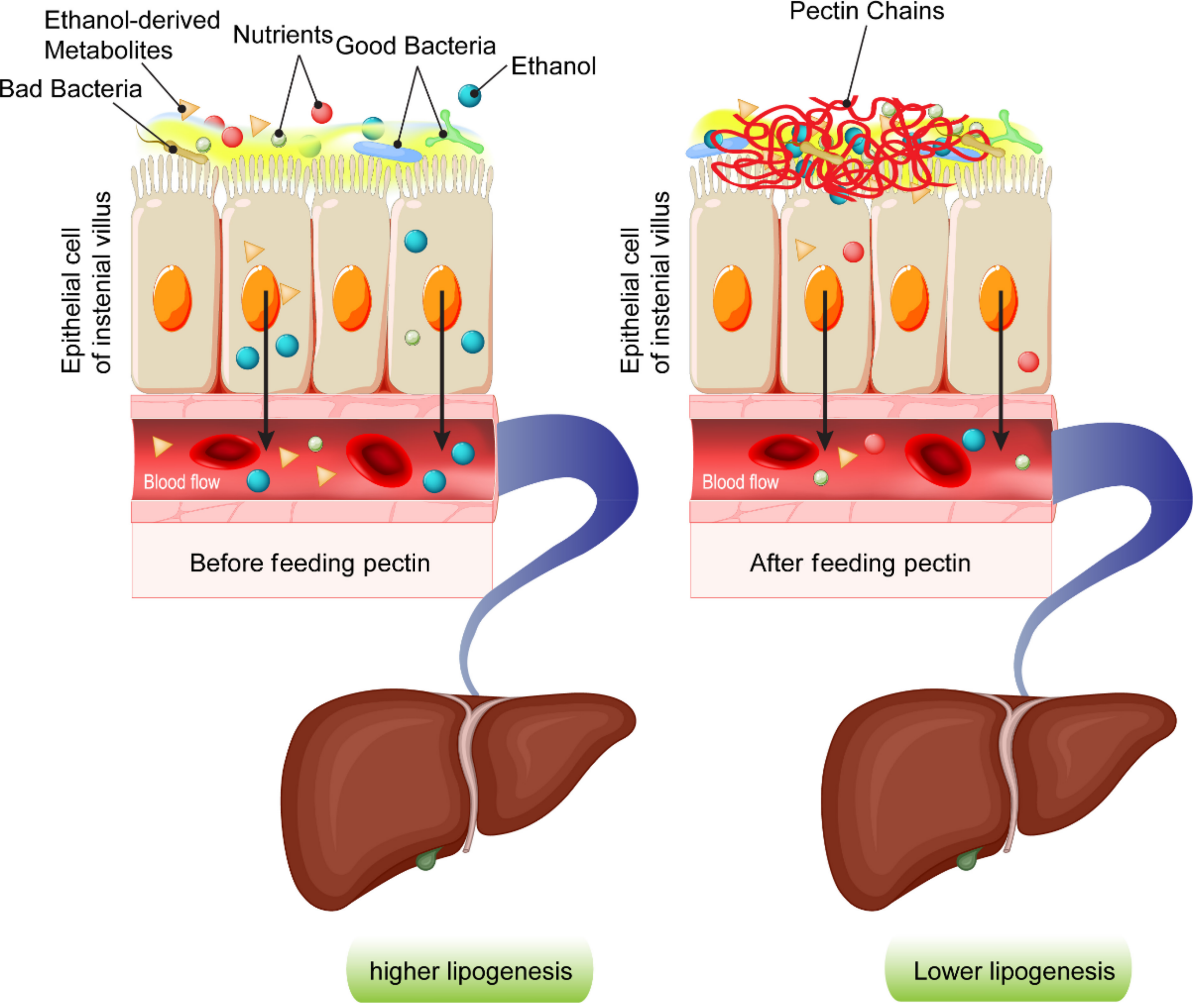
The role of pectin in enhancing the viscosity of intestinal mucus and its inhibitory impacts on absorption of ethanol or ethanol‐derived metabolites. These inhibitory effects ultimately reduce lipogenesis in the liver.

Ethanol intake interferes in fat metabolism and may lead to hypercholesterolemia (Ayaz & Alnahdi, 2018). Research has shown that oral administration of pectin at concentration of 0.2 g per 100 g of body weight in ethanol‐intoxicated rats resulted in a significant decrease in blood cholesterol, TG, and total lipid compared to control groups (Sheremeta & Haynuk, 2018). Pectin was also found to induce detoxification in mice after direct administration of ethanol into their stomachs. When the mice ingested pectin 30 min after ethanol administration, most of the pectin‐treated animals seemed to show no ethanol in their blood after testing with a gas chromatograph (Haynuk & Sheremeta, 2018). Pectin's partial ethanol neutralization and detoxification are associated with its carboxyl groups, which tend to interact with ethanol's hydroxyl groups. As a result of this interaction and the formation of a pectin complex, the viscosity of the intestinal fluid increases; this hinders the absorption of ethanol. The changes in viscosity of intestinal fluid adjacent to the brush border caused by pectin affect the epithelial absorption process, hindering the uptake of ethanol or ethanol‐derived metabolites across the intestine (Figure 4).

The viscosifying effects of dietary fiber ultimately reduce lipogenesis in the liver, leading to lower lipid accumulation. Moreover, pectin can interact with gastrointestinal components (bile salts) and the surrounding lipids in the intestine, which may induce their rapid loss into the feces. Thus, the binding of bile acids by pectin in the intestine contributes to more conversion of cholesterol to bile acids in the liver (Moslemi, 2021; Zhu, Hou, et al., 2017; Zhu, Sun, et al., 2017).

### 4.2 Aloe vera polysaccharides

Aloe vera is a perennial plant belonging to the *Aloeaceae* family. Aloe vera polysaccharides (AVPs) were shown to possess a rapid detoxification effect in the presence of reactive ethanol‐induced metabolites. For example, Cui et al. found that unpleasant hangover symptoms associated with ethanol intoxication were significantly reduced in mice fed with AVPs. The authors observed a better profile of antioxidant system in the serum of ethanol‐intoxicated mice fed with AVPs, with lower levels of total cholesterol and TG compared to control groups. In addition, mice fed with AVPs had lower accumulations of hepatic TG compared to those not fed with AVPs. AVP‐treated groups of mice also presented an increase in the specific activity of superoxide dismutase and glutathione levels, and AVPs reduced levels of the inflammatory mediators such as TNF‐α. AVPs also increased the expression of AMPK and PPAR‐α, which are involved in fatty acid beta‐oxidation in the liver. This led to accelerating fat degradation, resulting in a suppression of alcohol's intoxicating effects (Cui et al., 2014). The AVPs also effectively prevented the adsorption of bacterial LPS on the intestine, leading to their decreased presence in the blood; this makes it a potential candidate for treating alcohol‐induced endotoxemia. Although the influence of AVPs on ethanol‐induced metabolites remains unclear, the authors hypothesized that AVPs could exert a protective effect against alcohol‐induced liver injury by scavenging free radicals and inhibiting inflammatory responses (Kumar et al., 2019; Sierra‐Garcia et al., 2014).

### 4.3 Chito‐oligosaccharides

Chito‐oligosaccharides (COSs) are hydrolyzed products of chitosan that have been proven to be effective in alleviating ethanol‐induced hangover symptoms. Mice orally administered COSs (200 mg/kg) showed lower blood acetaldehyde levels compared to the control group. This could be due to the COS‐induced increased activity of ALDH (with no significant changes in mRNA expression), which is largely involved in detoxification of ethanol‐derived metabolites. The COSs may facilitate the attachment of ALDH to the acetaldehyde‐binding sites. It was also suggested that the individual administration of COSs increased the expression and phosphorylation activity of AMPK. Activated AMPK stimulated the formation of NAD^+^, which is required for ALDH enzymatic activity. AMPK activation is also followed by the phosphorylation of its substrate, acetyl‐CoA carboxylase 1, which plays an essential role in regulating fatty acid beta‐oxidation (Cho et al., 2010). Importantly, COSs could suppress oxidative stress by increasing the activity of glutathione peroxidase. Moreover, supplementation of COSs could significantly reverse the decline of hepatic glutathione levels caused by ethanol consumption (Bailey et al., 2001; Cho et al., 2010; Videla & Guerri, 2017).

### 4.4 Herbal medicine

Medicinal plants are appealing in toxicological science due to remediation of several toxicity‐induced diseases. They are popular around the world because of their least side effects in people compared to chemical medicines. *Camellia sinensis* is of popular medicinal plants in the world and its infusion is commonly used especially in Asia. With regard to anti‐hangover effect, promising results were observed by its extract in alleviation of tiredness and thirsty in the intoxicated individuals (Hwang & Kim, 2020). Extracts of *Houttuynia cordata*, *Nelumbo nucifera* G. leaf, and *Camellia sinensis* seed had ameliorating effect on hangover symptoms of intoxicated rats in the study of You et al. In their study, blood ethanol and acetaldehyde in the rats treated by herbal extract were lower than their levels in the control group. In addition, the herbal‐treated group showed an increased activity of liver ADH and ALDH, which helped in faster clearance of ethanol and acetaldehyde from the body (You et al., 2016). A formula containing *Viscum album* L., *Lycium chinense* L., *Inonotus obliquus*, and *Acanthopanax senticosus* H. could significantly increase blood ADH activity, suppress pro‐inflammatory cytokine, and cure the gastric damages caused by ethanol in rats fed with ethanol (Yoon et al., 2011). In study of Lee et al. (Lee, Kim, et al., 2009), hot water extract of medicinal plants of *Glycyrrhiza uralensis*, *Pueraria thunbergiana*, *Lycium chinense*, *Liriope platyphylla*, *Polypori umbellati* Polyporaceae, *Ixeris dentata*, *Acanthopanax sessiliflorus*, *Artemisia capillaris* Thunb, *Paeonia lactiflora* Pallas, *Hovenia dulcis* Thunb, *Plantago asiatica* L., and Panax ginseng (red) was prepared and fermented by *Lactobacillus delbruechii* ssp. *lactis*. Anti‐hangover effects of both fermented and non‐fermented herbal extracts were studied in the laboratory conditions. Interestingly, the authors observed a reduced ADH yet increased ALDH activity in the treated rats. Although effects of the fermented extract on activity of the enzymes were more significant than the non‐fermented extract (−62.63% vs. −20.22% for ADH; 280.17% vs. 173.20% for ALDH; Lee, Kim, et al., 2009). Similar study conducted by Noh et al. (2009) found that Dandelion juice could increase the concentrations of plasma ALDH and expression of catalase and glutathione reductase in males aged 24–28 years. Alleviation of hangover symptoms was also studied by Lieb and Schmitt (Lieb & Schmitt, 2020) in healthy adult individuals by administration of a formula containing *Malpighia glabra* fruit extract, *Opuntia ficus indica* fruit extract, *Ginkgo biloba* leaf extract, *Salix alba* leaf extract, and *Zingiber officinale* root extract enriched with vitamins and minerals. To understand the detoxification effects of plant extract, a second group delivered a supplement that contained vitamins and minerals in the absence of the plant extracts. In this study, the detoxification effects were significantly observed in the group that received the plant extracts but were not observed in another group supplied by only minerals and vitamins. On the basis of this observation, the authors concluded that restoration of electrolytes and vitamins to the body had no significant effect in alleviation of ethanol hangover symptoms and the main role was governed by the plant extracts presumably through activation of ADH and ALDH enzymes, inhibition of pro‐inflammatory mediators, and development of antioxidant activity (Lieb & Schmitt, 2020).

### 4.5 Minerals

Although Lieb and Schmitt (2020) found that the minerals may confer little or no effect in alleviating the ethanol‐induced side effects, their significant effects on repression of post‐alcohol symptoms have been reported by other scientists. In this contribution, selenium (Se), zinc (Zn), magnesium (Mg), copper (Cu), and vanadium (V) were reported to deliver the most effects in reducing the ethanol‐hangover symptoms (Min et al., 2010). Among these, selenium has a leading position to serve as a cofactor of glutathione peroxidase which is important in antioxidant reactions. Other than its role in antioxidant activities, it also contributes to expression of the genes responsible for production of immunomodulatory proteins. In addition, Mn, Cu, and Zn are also cofactors of the antioxidant enzyme of superoxide dismutase. Other minerals such as calcium (Ca), Zn, V, and Cu can also take part in oxidation–reduction reactions (Min et al., 2010). Mn blocks the N‐methyl‐d‐aspartate receptors (NMDRs) in the brain that is a target of ethanol leading to cognitive deficits in intoxicated individuals (Ron & Wang, 2009; Smith et al., 2009). Zn also contributes to NMDR and GABA receptors resulting in prevention of alcohol‐induced depression. However, mineral homeostasis is a determinant factor so that their consumption beyond the threshold may have adverse impact (Min et al., 2010). A recent study found that the highest activity of hepatic ADH and ALDH in mice treated by honey with different origins was observed for the samples containing the highest levels of Zn, Cu, and Fe, and relatively high amount of Mg (Guo et al., 2019).

### 4.6 Ginseng

Detoxification effects of red ginseng drink (containing 0.32 mg/mL red ginseng extract) was evaluated by Lee et al. It was found that the individuals who received the dosage of red ginseng drink had lower blood alcohol levels after 30, 45, and 60 min compared with the control group that received water after ethanol intake. The authors concluded that red ginseng possessed a long‐term and effective anti‐hangover effects associated with their detoxification potential due to the presence of antioxidant compounds that can suppress the oxidative stress developed after alcohol intake in the liver (Lee et al., 2014). In another study, linoleic acid was introduced as a bioactive compound in ginseng to modulate the activity of alcohol metabolism enzymes (Wang et al., 2016). Although it was found that that the presence of antioxidants in ginseng is more likely to relieve ethanol intoxication effects because of the susceptibility of the double bonds in linoleic acid to the oxidative reactions that occurred after ethanol intake (Warner et al., 2017). Similar studies presented a decreased blood alcohol concentration in participants who consumed a dosage of ethanol and fed with a formula containing *Hoveni dulcis* Thunb extract and ginseng‐berry extract (Park et al., 2019; Sharma et al., 2020). This outcome confirmed the detoxification effects of ginseng extract in ethanol‐intoxicated individuals.

### 4.7 *Hovenia dulcis* tree

Water extracts of oxidated leaf and branch of *H. dulcis* tree were examined for anti‐hangover potential and detoxification activity in mice before ethanol intake. The extracts were found to increase ADH and ALDH activity in the liver in the similar way to other food bioactive ingredients. The mixture of both extracts also increased the expression of ALDH2 (the major isozyme responsible for acetaldehyde clearance) and SOD2 (an antioxidant enzyme responsible for breaking down superoxide radicals) in the liver. Nitration of tyrosine is a side effect reaction that occurs under ethanol drinking and leads to deformation of proteinaceous structures including catalase responsible for alcohol metabolism. Interestingly, tyrosine nitration was suppressed significantly in the mice fed by a mixture of oxidated leaf and branch of *H. dulcis*, in which the polyphenol compounds present in the extracts played a great role. The highest anti‐nitration effect was related to the extract prepared by branch of *H. dulcis* tree due to the activity of catechin as the abundant flavonoid. In addition, the antioxidant potency of the extracts could successfully inhibit glutathione depletion in the liver of the treated mice (Je et al., 2021). The favorable activity of *H. dulcis* extract might also be due to its effect on increased level of IL‐10 as anti‐inflammatory compound and decreased production of IL‐6 as pro‐inflammatory agent (Kim et al., 2017). Furthermore, *H. dulcis* contains a flavonoid known as dihydromyricetin that able to interact with GABA receptors and block alcohol intoxication by which some hangover symptoms are suppressed (Wang et al., 2016). Similar detoxification role was observed by the administration of theracurmin drink containing *H. dulcis* fruit stalk and theracurmin (highly bioavailable curcumin) extracts. This bioactive drink could successfully increase GSH level in the ethanol‐fed rats by using its antioxidant activity (Kang et al., 2021). Other than functionality of the bioactive compounds naturally present in the plants, their bioprocessing can exacerbate their potential detoxification effects. For example, greater anti‐hangover effects were observed for fermented *H. dulcis* Thunb fruit extract in reduction of blood alcohol and acetaldehyde compared with non‐fermented extract (Choi et al., 2014).

### 4.8 Other food components

In addition to the functional foods and bioactive food compounds mentioned earlier, several other food‐based components have been found to alleviate the hangover symptoms of alcoholism with their detoxification effects. It is notable that some of these compounds are commercially available. A liquid‐based formula named Oh!K, composed of ginger, green tea, turmeric, and pepper, could successfully diminish post‐alcohol disorders in intoxicated drinkers (Sreeraj Gopi et al., 2014). Another herbal formula, with the commercial name of “PartySmart,” was found to prevent ethanol‐induced hangovers by increasing the activities of ADL and ALDH in the liver, resulting in the rapid breakdown of acetaldehydes (Pordie, 2015). “DotShot,” a product containing curcumin and lemon as its principal ingredients, was also reported to be efficient in the management of alcohol hangover by increasing the activity of ALDH (Harisha, 2018).

The mixture of glutathione‐enriched yeast extract and rice embryo/soybean extract fermented by *Bacillus subtilis* Natto was examined for alleviation of hangover symptoms by Lee et al. In their study, one group of rats was subjected to a 2‐week pretreatment by the mixture and another group was fed by the mixture 1 h after ethanol administration. The authors concluded that bioactive compounds including thiamin and Zn present in the mixture inhibited the absorption of ethanol in the intestine or their antioxidant activities prevented the body from oxidative damages and consequent symptoms. In addition, the expression of CYP2E1 was decreased in both groups suggesting less oxidative stress in the extract‐treated rats. In addition, increased expression and activity of ADH and ALDH in the liver by the mixture was of great importance (Lee, Song, et al., 2009). Similarly, the blood concentration of ethanol and acetaldehyde in alcohol‐intoxicated rats treated by bioprocessed black rice bran and glutathione‐enriched yeast extract were significantly reduced. It was concluded that antioxidant potential of ɤ‐oryzanol in the rice bran and the role of cysteine in glutathione (i.e., a tripeptide composed of glutamate–cysteine–glycine) toward acetaldehyde inactivation were important protective factors (Kim et al., 2021).

Increased ADH activity by food sources was also approved in the study of Lee and Bae (2011), through which ADH activity was increased by 63% in vitro in the presence of ethanolic extract of mulberry compared to a commercial detox drink. Similar results with respect to the stimulatory effect of propolis on hepatic ADH and ALDH activity after ethanol administration were reported by Lee and Park (Lee & Park, 2021). Persimmon juice fermented by *Lactobacillus* species could increase ADH and ALDH activity in vitro owing to the activity of GABA produced by the bacterial fermentation and other bioactive compounds in the fruit (Zhou et al., 2019). It was reported that GABA may inhibit intestinal absorption of ethanol and stimulate the expression and the activity of alcohol metabolizing enzymes (Boby et al., 2021; Cha et al., 2011). The last effect might be due to the antioxidant activity of GABA (Zhu et al., 2019). It has been reported that oxidative stress and progress of lipid peroxidation decrease the activity of ALDH and lead to development of hangover symptoms in drinkers (Kim et al., 2018; Min et al., 2010). Therefore, natural antioxidants in foodstuff have positive effects on ADH and ALDH activity by control of oxidation reactions (Lee, Isse, et al., 2013; Seo et al., 2014). On the other hand, ALDH2 stimulates GABA synthesis from acetate through TCA cycle by which analgesic effect of ethanol is perceived (Jin, Cao, et al., 2021; Jin, Cinar, et al., 2021). Therefore, the development of acetaldehyde metabolism in favor of GABA synthesis is another route in suppression of hangover symptoms.

Protective effects of epigallocatechin‐3‐gallate (EGCG), the polyphenols from green tea, in alleviating alcohol hangover symptoms have also been approved. Czech and Seki (2020) reported that EGCG may be used as a promising therapy for alcohol‐induced disorders. EGCG supplementation in rats can regulate toll‐like receptors (TLRs) and has been shown to suppress the elevation of intrahepatic TLRs (TLR2, TLR4, and TLR9) in the Kupffer cells by inducing the production of immunomodulatory cytokines such as interleukin‐10 (IL‐10) (Liu et al., 2014). Diosmin (Diosmetin‐7‐O‐rutinoside), a naturally occurring citrus flavone, was observed to induce similar preventive effects in the treatment of ethanol‐intoxicated rats by alleviating ethanol‐induced hepatic and gastric lesions. These beneficial outcomes are partly due to its remarkable antioxidant and anti‐inflammatory activities, which reduce oxidative stress in the gastric mucosa (Arab et al., 2015). Cuevas et al. found that grapeseed extract could attenuate the development of ethanol‐induced gastric ulcerative lesions and MDA levels (Cuevas et al., 2011).

An early in vivo trial report by Uto‐Kondo et al. studied the detoxification and hepatoprotective effects of the extract form of shiitake, a popular edible mushroom in Japan and China, and its roles in reducing ethanol‐induced metabolites. The authors found that shiitake extract, which contains a large amount of lentinic acid, could suppress the absorption of both ethanol and acetaldehyde from the stomach and jejunum, likely due to its property of slowing the peristaltic movements in the GI tract. Moreover, the administration of shiitake extract to rats was shown to decrease blood ethanol or acetaldehyde concentrations owing to a rise in the enzymatic activities of cytosolic ADH, cytosolic ALDH, and mitochondrial ALDH (ALDH2) in the stomach and liver (Uto‐Kondo et al., 2020). The same authors also investigated the anti‐hangover effects of garlic extract, which contains high concentrations of S‐allyl‐L‐cysteine sulfoxide. The results suggested that garlic extract relieved intoxication and hangover by reducing intestinal alcohol and acetaldehyde absorption and metabolism. The garlic extract showed the most significant stimulatory effects on ALDH2 (Uto‐Kondo et al., 2018).

Srinivasan et al. studied the anti‐hangover effectiveness and efficiency of several selected food commodities, including fruits, vegetables, cereals, pulses, dairy products, and spices, after alcohol consumption. The results showed that pear, sweet lime, and coconut water developed anti‐hangover effects, which were validated by the evidence of increased ADH and ALDH activity in vitro. In contrast, the authors recommended against consuming coffee after ethanol ingestion because of its property of prolonging the time needed to clear acetaldehyde from the body as a result of decreased activity of both ADH and ALDH. Interestingly, the authors could not find any correlation between the antioxidant capacity and the activity of ADH and ALDH (Srinivasan et al., 2019). Despite this lack of relationship, the positive impacts of antioxidants in the suppression of oxidative stress after alcohol consumption should not be ignored (Hipolito et al., 2015; Lee, Kim, & Min, 2013; Ozkol et al., 2017; Srinivasan et al., 2019).

## 5 CONCLUSION

As highlighted throughout this review, ethanol consumption is primarily linked to gastrointestinal malabsorption of micronutrients, in particular water‐soluble vitamins, and their deficiencies in the body. Other than food supplementation in compensation for the not‐absorbed and/or lost nutrients through ethanol drinking, several dietary food components were identified as detoxifying agent that could influence alcohol metabolism, and thereby potentially alleviate the development of alcohol hangover symptoms. This critical review comprehensively discussed those various food components including natural sources and several commercial plant‐based products that may suppress the severity of alcohol hangover symptoms via their antioxidant and/or anti‐inflammatory properties. This review also explored the proposed mechanism for how some of these food‐based products might alleviate alcohol hangover symptoms. As detailed, these natural compounds mainly reduced the levels of blood alcohol metabolites by increasing the activity of ADH and ALDH. The insights gained through this review may assist clinicians in understanding and managing alcohol‐associated diseases. Given the current intense research activities in exploration of natural therapeutic prospects, we anticipate several breakthroughs in the development of natural medications to help drinkers with severe hangover symptoms.

## AUTHOR CONTRIBUTIONS

**Masoumeh Moslemi:** Conceptualization (lead); investigation (lead); project administration (lead); software (lead); supervision (lead); writing – original draft (lead); writing – review and editing (lead). **Behrooz Jannat:** Supervision (equal). **Maryam Mahmoudzadeh:** Project administration (equal). **Mehran Ghasemlou:** Investigation (supporting); project administration (supporting); supervision (supporting); writing – original draft (supporting); writing – review and editing (lead). **Abdol‐Samad Abedi:** Writing – original draft (equal); writing – review and editing (equal).

## CONFLICT OF INTEREST STATEMENT

The authors declare that they have no conflict of interest.

## Data Availability

The data that support the findings of this study are available on request from the corresponding author.

## References

[fsn33520-bib-0001] Alzeer, J. , & Abou Hadeed, K. (2016). Ethanol and its halal status in food industries. Trends in Food Science & Technology, 58, 14–20.

[fsn33520-bib-0002] Arab, H. H. , Salama, S. A. , Omar, H. A. , Arafa, E. S. A. , & Maghrabi, I. A. (2015). Diosmin protects against ethanol‐induced gastric injury in rats: Novel anti‐ulcer actions. PLoS One, 10, e0122417. 25821971 10.1371/journal.pone.0122417 PMC4378914

[fsn33520-bib-0003] Ayaz, N. O. , & Alnahdi, H. S. (2018). Potential impact of Panax ginseng against ethanol induced hyperlipidemia and cardiac damage in rats. Pakistan Journal of Pharmaceutical Sciences, 31(3), 927–932. 29716875

[fsn33520-bib-0004] Bailey, S. M. , Patel, V. B. , Young, T. A. , Asayama, K. , & Cunningham, C. C. (2001). Chronic ethanol consumption alters the glutathione/glutathione peroxidase‐1 system and protein oxidation status in rat liver. Alcoholism: Clinical and Experimental Research, 25, 726–733. 11371722

[fsn33520-bib-0005] Boby, N. , Lee, E. B. , Abbas, M. A. , Park, N. H. , Lee, S. P. , Ali, M. S. , Lee, S. J. , & Park, S. C. (2021). Ethanol‐induced hepatotoxicity and alcohol metabolism regulation by GABA‐enriched fermented Smilax China root extract in rats. Food, 10(10), 2381. 10.3390/foods10102381 PMC8535858 34681429

[fsn33520-bib-0006] Bode, C. , & Bode, J. C. (2003). Effect of alcohol consumption on the gut. Best Practice & Research Clinical Gastroenterology, 17, 575–592. 12828956 10.1016/s1521-6918(03)00034-9

[fsn33520-bib-0007] Burton, R. , & Sheron, N. (2018). No level of alcohol consumption improves health. The Lancet, 392, 987–988. 10.1016/S0140-6736(18)31571-X 30146328

[fsn33520-bib-0008] Butts, M. , Singh Paulraj, R. , Haynes, J. , Arthur, S. , Singh, S. , & Sundaram, U. (2019). Moderate alcohol consumption inhibits sodium‐dependent glutamine co‐transport in rat intestinal epithelial cells in vitro and ex vivo. Nutrients, 11, 2516. 31635319 10.3390/nu11102516 PMC6835445

[fsn33520-bib-0009] Cederbaum, A. I. (2010). Role of CYP2E1 in ethanol‐induced oxidant stress, fatty liver and hepatotoxicity. Digestive Diseases, 28, 802–811. 21525766 10.1159/000324289 PMC3211519

[fsn33520-bib-0010] Cederbaum, A. I. (2012). Alcohol metabolism. Clinics in Liver Disease, 16, 667–685. 23101976 10.1016/j.cld.2012.08.002 PMC3484320

[fsn33520-bib-0011] Cha, J. Y. , Lee, B. J. , Je, J. Y. , Kang, Y. M. , Kim, Y. M. , & Cho, Y. S. (2011). GABA‐enriched fermented Laminaria japonica protects against alcoholic hepatotoxicity in Sprague‐Dawley rats. Fisheries and Aquatic Sciences, 14, 79–88.

[fsn33520-bib-0012] Charlet, K. , & Heinz, A. (2017). Harm reduction‐ a systematic review on effects of alcohol reduction on physical and mental symptoms. Addiction Biology, 22, 1119–1159. 27353220 10.1111/adb.12414

[fsn33520-bib-0013] Cheraghali, F. , Shojaee‐Aliabadi, S. , Hosseini, S. M. , Mirmoghtadaie, L. , Mortazavian, A. M. , Ghanati, K. , Abedi, A. S. , & Moslemi, M. (2018). Characterization of microcapsule containing walnut (*Juglans regia* L.) green husk extract as preventive antioxidant and antimicrobial agent. International journal of Preventive Medicine, 9, 101. 30598739 10.4103/ijpvm.IJPVM_308_18 PMC6259432

[fsn33520-bib-0014] Cho, S. Y. , Yun, J. W. , Park, P. J. , Sohn, J. H. , Seo, D. B. , Lim, K. M. , Kim, W. G. , & Lee, S. J. (2010). Effects of chitooligosaccharide lactate salt on activity of acetaldehyde dehydrogenase. Journal of Medicinal Food, 13, 1061–1068. 20828325 10.1089/jmf.2009.1323

[fsn33520-bib-0015] Choi, J. Y. , Kim, J. H. , Kim, G. , Kim, C. K. , & Choi, M. S. (2014). Effect of fermented *Hovenia dulcis* Thunb fruit water extract on biomarker for liver injury and body weight changes in rats given oral administration of ethanol. Korean Journal of Food Preservation, 21, 412–420.

[fsn33520-bib-0016] Cuevas, V. M. , Calzado, Y. R. , Guerra, Y. P. , Yera, A. O. , Despaigne, S. J. , Ferreiro, R. M. , & Quintana, D. C. (2011). Effects of grape seed extract, vitamin C, and vitamin E on ethanol‐ and aspirin‐induced ulcers. Advances in Pharmacological Sciences, 2011, 740687. 22162675 10.1155/2011/740687 PMC3226337

[fsn33520-bib-0017] Cui, Y. , Ye, Q. , Wang, H. , Li, Y. , Yao, W. , & Qian, H. (2014). Hepatoprotective potential of Aloe vera polysaccharides against chronic alcohol‐induced hepatotoxicity in mice. Journal of the Science of Food and Agriculture, 94, 1764–1771. 24272968 10.1002/jsfa.6489

[fsn33520-bib-0018] Czech, T. Y. , & Seki, E. (2020). Kupffer cell TLR2/3 signaling: A pathway for EGCG amelioration of ethanol‐induced hepatic injury. Cellular and Molecular Gastroenterology and Hepatology, 9, 187–188. 31669157 10.1016/j.jcmgh.2019.10.001 PMC6926269

[fsn33520-bib-0019] Di Castelnuovo, A. , Costanzo, S. , Bonaccio, M. , Rago, L. , De Curtis, A. , Persichillo, M. , Bracone, F. , Olivieri, M. , Cerletti, C. , & Donati, M. B. (2017). Moderate alcohol consumption is associated with lower risk for heart failure but not atrial fibrillation. JACC: Heart Failure, 5, 837–844. 29032141 10.1016/j.jchf.2017.08.017

[fsn33520-bib-0020] Fernando, M. (2017). Evidence for benefit of low‐dose alcohol. Canadian Family Physician, 63, 916–917. PMC5729136 29237628

[fsn33520-bib-0021] Gonzalez‐Reimers, E. , Fernandez‐Rodriguez, C. M. , Martin‐Gonzalez, M. C. , Hernandez‐Betancor, I. , Abreu‐Gonzalez, P. , Vega‐Prieto, M. J. D. L. , Elvira‐Cabrera, O. , & Santolaria‐Fernandez, F. (2014). Antioxidant vitamins and brain dysfunction in alcoholics. Alcohol and Alcoholism, 49, 45–50. 24070686 10.1093/alcalc/agt150

[fsn33520-bib-0022] Gonzalez‐Reimers, E. , Quintero‐Platt, G. , Martin‐Gonzalez, M. C. , Romero‐Acevedo, L. , & Santolaria‐Fernandez, F. (2017). Antioxidant vitamins and brain dysfunction in alcoholics. In Addictive substances and neurological disease (pp. 163–179). Elsevier Science.

[fsn33520-bib-0023] Gopi, S. , George, R. , Thankachen, R. U. , Sriraam, V. , & Abirami, S. (2014). Studies on the effectiveness and safety of anti‐hangover drink (Oh! K) in reducing cocktail (alcohol) induced hangover symptoms in adult male social drinkers. International Journal of Herbal Medicine, 2, 115–117.

[fsn33520-bib-0024] Guengerich, P. F. , & Avadhani, N. G. (2018). Roles of cytochrome P450 in metabolism of ethanol and carcinogens. In Alcohol and Cancer (pp. 15–35). Springer New York. 10.1007/978-3-319-98788-0_2 PMC6371814 30362088

[fsn33520-bib-0025] Guo, P. , Deng, Q. , & Lu, Q. (2019). Anti‐alcoholic effects of honeys from different floral origins and their correlation with honey chemical compositions. Food Chemistry, 286, 608–615. 30827653 10.1016/j.foodchem.2019.02.058

[fsn33520-bib-0026] Harisha, S. (2018). A study to evaluate the safety and efficacy of ‘dotshot’ in the treatment of hangover due to alcohol intoxication. European Journal of Pharmaceutical and Medical Research, 5, 680–689.

[fsn33520-bib-0027] Haynuk, M. , & Sheremeta, L. (2018). Detoxifying effect of apple pectin under experimental acute alcohol intoxication. Medical and Clinical Chemistry, 2, 72–76.

[fsn33520-bib-0028] Haynuk, M. , & Sheremeta, L. (2019). Тhe apple pectin influence upon the liver histological structure and the activity of lipid peroxidation in experimental acute alcohol intoxication. Pharma Innovation, 8, 590–593.

[fsn33520-bib-0029] Hipolito, U. V. , Callera, G. E. , Simplicio, J. A. , De Martinis, B. S. , Touyz, R. M. , & Tirapelli, C. R. (2015). Vitamin C prevents the endothelial dysfunction induced by acute ethanol intake. Life Sciences, 141, 99–107. 26386369 10.1016/j.lfs.2015.09.006

[fsn33520-bib-0030] Husain, K. , Ferder, L. , Ansari, R. A. , & Lalla, J. (2011). Chronic ethanol ingestion induces aortic inflammation/oxidative endothelial injury and hypertension in rats. Human & Experimental Toxicology, 30, 930–939. 20921064 10.1177/0960327110384520 PMC4651422

[fsn33520-bib-0031] Hwang, J. H. , & Kim, M. Y. (2020). Natural herbal extract complex induces the degradation of alcohol and acetaldehyde and reduces the breath alcohol concentration. The Journal of the Convergence on Culture Technology, 6, 381–392.

[fsn33520-bib-0032] Hyun, J. , Han, J. , Lee, C. , Yoon, M. , & Jung, Y. (2021). Pathophysiological aspects of alcohol metabolism in the liver. International Journal of Molecular Sciences, 22, 5717. 34071962 10.3390/ijms22115717 PMC8197869

[fsn33520-bib-0033] Je, J. , Song, M. , Baek, J. H. , Kang, J. S. , Chung, H. J. , Lee, K. , Park, S. W. , & Kim, H. J. (2021). Combined water extracts from oxidation‐treated leaves and branches of *Hovenia dulcis* has anti‐hangover and liver protective effects in binge alcohol intake of male mice. Nutrients, 13, 4404. 34959956 10.3390/nu13124404 PMC8707081

[fsn33520-bib-0034] Jiang, Y. , Zhang, T. , Kusumanchi, P. , Han, S. , Yang, Z. , & Liangpunsakul, S. (2020). Alcohol metabolizing enzymes, microsomal ethanol oxidizing system, cytochrome P450 2E1, catalase, and aldehyde dehydrogenase in alcohol‐associated liver disease. Biomedicine, 8, 50. 10.3390/biomedicines8030050 PMC7148483 32143280

[fsn33520-bib-0035] Jin, S. , Cao, Q. , Yang, F. , Zhu, H. , Xu, S. , Chen, Q. , Wang, Z. , Lin, Y. , Cinar, R. , & Pawlosky, R. J. (2021). Brain ethanol metabolism by astrocytic ALDH2 drives the behavioural effects of ethanol intoxication. Nature Metabolism, 3, 337–351. 10.1038/s42255-021-00357-z PMC8294184 33758417

[fsn33520-bib-0036] Jin, S. , Cinar, R. , Hu, X. , Lin, Y. , Luo, G. , Lovinger, D. M. , Zhang, Y. , & Zhang, L. (2021). Spinal astrocyte aldehyde dehydrogenase‐2 mediates ethanol metabolism and analgesia in mice. British Journal of Anaesthesia, 127, 296–309. 33934892 10.1016/j.bja.2021.02.035 PMC8362281

[fsn33520-bib-0037] Kamran, U. , Towey, J. , Khanna, A. , Chauhan, A. , Rajoriya, N. , & Holt, A. (2020). Nutrition in alcohol‐related liver disease: Physiopathology and management. World Journal of Gastroenterology, 26, 2916–2930. 32587439 10.3748/wjg.v26.i22.2916 PMC7304106

[fsn33520-bib-0038] Kang, N. E. , Oh, Y. S. , Yeo, H. K. , Baik, H. W. , & Jang, S. E. (2021). Effects of a health drink containing the extract of the *Hovenia Dulcis* fruit stalk and theracurmin, on ethanol‐induced hangover. Journal of the Korean Society of Food Culture, 36, 563–570.

[fsn33520-bib-0039] Kawaratani, H. , Tsujimoto, T. , Douhara, A. , Takaya, H. , Moriya, K. , Namisaki, T. , Noguchi, R. , Yoshiji, H. , Fujimoto, M. , & Fukui, H. (2013). The effect of inflammatory cytokines in alcoholic liver disease. Mediators of Inflammation, 2013, 495156. 24385684 10.1155/2013/495156 PMC3872233

[fsn33520-bib-0040] Khan, I. , Bhardwaj, M. , Shukla, S. , Min, S. H. , Choi, D. K. , Bajpai, V. K. , Huh, Y. S. , & Kang, S. C. (2019). Carvacrol inhibits cytochrome P450 and protects against binge alcohol‐induced liver toxicity. Food and Chemical Toxicology, 131, 110582. 31220535 10.1016/j.fct.2019.110582

[fsn33520-bib-0041] Kim, H. , Kim, Y. J. , Jeong, H. Y. , Kim, J. Y. , Choi, E. K. , Chae, S. W. , & Kwon, O. (2017). A standardized extract of the fruit of *Hovenia dulcis* alleviated alcohol‐induced hangover in healthy subjects with heterozygous ALDH2: A randomized, controlled, crossover trial. Journal of Ethnopharmacology, 209, 167–174. 28750942 10.1016/j.jep.2017.07.028

[fsn33520-bib-0042] Kim, M. J. , Lim, S. W. , Kim, J. H. , Choe, D. J. , Kim, J. I. , & Kang, M. J. (2018). Effect of mixed fruit and vegetable juice on alcohol hangovers in healthy adults. Preventive Nutrition and Food Science, 23(1), 1–7. 29662841 10.3746/pnf.2018.23.1.1 PMC5894779

[fsn33520-bib-0043] Kim, S. P. , Lee, J. R. , Kwon, K. S. , Jang, Y. J. , Kim, J. , Yu, K. H. , Lee, S. Y. , & Friedman, M. (2021). A bioprocessed black rice bran glutathione‐enriched yeast extract protects rats and mice against alcohol‐induced hangovers. Food and Nutrition Sciences, 12, 223–238.

[fsn33520-bib-0044] Kumar, R. , Singh, A. K. , Gupta, A. , Bishayee, A. , & Pandey, A. K. (2019). Therapeutic potential of Aloe vera‐ a miracle gift of nature. Phytomedicine, 60, 152996. 31272819 10.1016/j.phymed.2019.152996

[fsn33520-bib-0045] Lee, E. J. , & Bae, J. H. (2011). Study on the alleviation of an alcohol induced hangover and the antioxidant activity by mulberry fruit. The Korean Journal of Food and Nutrition, 24, 204–209.

[fsn33520-bib-0046] Lee, H. S. , Isse, T. , KawaFmoto, T. , Baik, H. W. , Park, J. Y. , & Yang, M. (2013). Effect of Korean pear (Pyruspyrifolia cv. Shingo) juice on hangover severity following alcohol consumption. Food and Chemical Toxicology, 58, 101–106. 23587660 10.1016/j.fct.2013.04.007

[fsn33520-bib-0047] Lee, H. S. , Song, J. , Kim, T. M. , Joo, S. S. , Park, D. , Jeon, J. H. , Shin, S. , Park, H. K. , Lee, W. K. , & Ly, S. Y. (2009). Effects of a preparation of combined glutathione‐enriched yeast and rice embryo/soybean extracts on ethanol hangover. Journal of Medicinal Food, 12, 1359–1367. 20041794 10.1089/jmf.2008.1367

[fsn33520-bib-0048] Lee, K. S. , Kim, G. H. , Seong, B. J. , Kim, H. H. , Kim, M. Y. , & Kim, M. R. (2009). Effects of aqueous medicinal herb extracts and aqueous fermented extracts on alcohol‐metabolizing enzyme activities. Korean Journal of Food Preservation, 16, 259–265.

[fsn33520-bib-0049] Lee, M. H. , Kwak, J. H. , Jeon, G. , Lee, J. W. , Seo, J. H. , Lee, H. S. , & Lee, J. H. (2014). Red ginseng relieves the effects of alcohol consumption and hangover symptoms in healthy men: A randomized crossover study. Food & Function, 5, 528–534. 24458173 10.1039/c3fo60481k

[fsn33520-bib-0050] Lee, S. , & Park, Y. S. (2021). Effect of water‐soluble propolis administration on the ethanol‐induced hangover in rats. Food Science and Biotechnology, 30, 455–463. 33868756 10.1007/s10068-020-00869-6 PMC8017057

[fsn33520-bib-0051] Lee, S. J. , Kim, S. Y. , & Min, H. (2013). Effects of vitamin C and E supplementation on oxidative stress and liver toxicity in rats fed a low‐fat ethanol diet. Nutrition Research and Practice, 7, 109–114. 23610603 10.4162/nrp.2013.7.2.109 PMC3627927

[fsn33520-bib-0052] Lieb, B. , & Schmitt, P. (2020). Randomised double‐blind placebo‐controlled intervention study on the nutritional efficacy of a food for special medical purposes (FSMP) and a dietary supplement in reducing the symptoms of veisalgia. BMJ Nutrition, Prevention & Health, 3, 31–39. 10.1136/bmjnph-2019-000042 PMC7664490 33235969

[fsn33520-bib-0053] Liu, D. , Zhang, X. , Jiang, L. , Guo, Y. , & Zheng, C. J. (2014). Epigallocatechin‐3‐gallate (EGCG) attenuates concanavalin A‐induced hepatic injury in mice. Acta Histochemica, 116, 654–662. 24373695 10.1016/j.acthis.2013.12.002

[fsn33520-bib-0054] Lu, J. , Zhu, X. , Zhang, C. , Lu, F. , Lu, Z. , & Lu, Y. (2020). Co‐expression of alcohol dehydrogenase and aldehyde dehydrogenase in *Bacillus subtilis* for alcohol detoxification. Food and Chemical Toxicology, 135, 110890. 31628963 10.1016/j.fct.2019.110890

[fsn33520-bib-0055] Min, J. A. , Lee, K. , & Kim, D. J. (2010). The application of minerals in managing alcohol hangover: A preliminary review. Current Drug Abuse Reviews, 3, 110–115. 20712595 10.2174/1874473711003020110

[fsn33520-bib-0056] Minzer, S. , Losno, R. A. , & Casas, R. (2020). The effect of alcohol on cardiovascular risk factors: Is there new information? Nutrients, 12, 912. 32230720 10.3390/nu12040912 PMC7230699

[fsn33520-bib-0057] Miyazaki, A. , Sano, M. , Fukuwatari, T. , & Shibata, K. (2012). Effects of ethanol consumption on the B‐group vitamin contents of liver, blood and urine in rats. British Journal of Nutrition, 108, 1034–1041. 22172166 10.1017/S0007114511006192

[fsn33520-bib-0058] Mohn, E. S. , & Johnson, E. J. (2015). Nutrient absorption in the human gastrointestinal tract. In C. M. Sabliov , H. Chen , & R. Y. Yada (Eds.), Nanotechnology and functional foods: Effective delivery of bioactive ingredients. Wiley.

[fsn33520-bib-0059] Moslemi, M. (2021). Reviewing the recent advances in application of pectin for technical and health promotion purposes: From laboratory to market. Carbohydrate Polymers, 254, 117324. 33357885 10.1016/j.carbpol.2020.117324

[fsn33520-bib-0060] Moslemi, M. , Hosseini, H. , Erfan, M. , Mortazavian, A. M. , Mazaheri Nezhad Fard, R. , Neyestani, T. R. , & Komeyli, R. (2014). Characterisation of spray‐dried microparticles containing iron coated by pectin/resistant starch. International Journal of Food Science & Technology, 49, 1736–1742.

[fsn33520-bib-0061] Moslemi, M. , Hosseini, H. , Neyestani, T. R. , Akramzadeh, N. , & Mazaheri Nezhad Fard, R. (2018). Effects of non‐digestive polymers used in iron encapsulation on calcium and iron apparent absorption in rats fed by infant formula. Journal of Trace Elements in Medicine and Biology, 50, 393–398. 30262310 10.1016/j.jtemb.2018.08.004

[fsn33520-bib-0062] Moslemi, M. , Kheirandish, M. , Mazaheri Nezhad Fard, R. , Hosseini, H. , Jannat, B. , Mofid, V. , Moghaddam, A. , & Karimian, N. (2020). National food policies in the Islamic Republic of Iran aimed at control and prevention of noncommunicable diseases. Eastern Mediterranean Health Journal, 26, 1556–1564. 33355396 10.26719/emhj.20.024

[fsn33520-bib-0063] Niemela, O. (2001). Distribution of ethanol‐induced protein adducts in vivo: Relationship to tissue injury. Free Radical Biology and Medicine, 31, 1533–1538. 11744326 10.1016/s0891-5849(01)00744-4

[fsn33520-bib-0064] Noh, K. H. , Jang, J. H. , Kim, J. J. , Shin, J. H. , Kim, D. K. , & Song, Y. S. (2009). Effect of dandelion juice supplementation on alcohol‐induced oxidative stress and hangover in healthy male college students. Journal of the Korean Society of Food Science and Nutrition, 38, 683–693.

[fsn33520-bib-0065] O'Keefe, E. L. , Di Nicolantonio, J. J. , O'Keefe, J. H. , & Lavie, C. J. (2018). Alcohol and CV health: Jekyll and Hyde J‐curves. Progress in Cardiovascular Diseases, 61, 68–75. 29458056 10.1016/j.pcad.2018.02.001

[fsn33520-bib-0066] Ozkol, H. , Bulut, G. , Balahoroglu, R. , Tuluce, Y. , & Ozkol, H. U. (2017). Protective effects of selenium, N‐acetylcysteine and vitamin E against acute ethanol intoxication in rats. Biological Trace Element Research, 175, 177–185. 27250492 10.1007/s12011-016-0762-8

[fsn33520-bib-0067] Park, N. H. , Lee, J. O. , & Cho, I. H. (2019). Effects of mixed supplementation on *Hoveni dulcis* Thunb extracts and ginseng‐berry extracts on hangover curves. The Journal of the Convergence on Culture Technology, 5, 359–367.

[fsn33520-bib-0068] Persson, E. C. , Schwartz, L. M. , Park, Y. , Trabert, B. , Hollenbeck, A. R. , Graubard, B. I. , Freedman, N. D. , & Mc Glynn, K. A. (2013). Alcohol consumption, folate intake, hepatocellular carcinoma, and liver disease mortality. Cancer Epidemiology and Prevention Biomarkers, 22, 415–421. 10.1158/1055-9965.EPI-12-1169 PMC3596467 23307533

[fsn33520-bib-0069] Pordie, L. (2015). Hangover free! The social and material trajectories of PartySmart. Anthropology & Medicine, 22, 34–48. 25641683 10.1080/13648470.2015.1004773

[fsn33520-bib-0070] Rajendram, R. , & Preedy, V. R. (2005). Effect of alcohol consumption on the gut. Digestive Diseases, 23, 214–221. 16508285 10.1159/000090168

[fsn33520-bib-0071] Rekve, D. , Banatvala, N. , Karpati, A. , Tarlton, D. , Westerman, L. , Sperkova, K. , Casswell, S. , Duennbier, M. , Rojhani, A. , & Bakke, Ø. (2019). Prioritising action on alcohol for health and development. BMJ, 367, 16162. 10.1136/bmj.l6162 31810905

[fsn33520-bib-0072] Ron, D. , & Wang, J. (2009). The NMDA receptor and alcohol addiction. In Biology of the NMDA Receptor (pp. 59–78). CRC Press. 21204417

[fsn33520-bib-0073] Sæther, S. M. M. , Knapstad, M. , Askeland, K. G. , & Skogen, J. C. (2019). Alcohol consumption, life satisfaction and mental health among Norwegian college and university students. Addictive Behaviors Reports, 10, 100216. 31692685 10.1016/j.abrep.2019.100216 PMC6806384

[fsn33520-bib-0074] Seitz, H. , & Mueller, S. (2014). Alcohol: Metabolism, toxicity and its impact on nutrition. In R. Dulbecco (Ed.), Encyclopedia of human biology. Academic Press.

[fsn33520-bib-0075] Seitz, H. K. , & Suter, P. M. (2002). Ethanol toxicity and nutritional status. In F. N. Kotsonis , M. Mackey , & J. J. Hjelle (Eds.), Nutritional toxicology. Taylor & Francis.

[fsn33520-bib-0076] Seo, J. Y. , Kim, S. S. , & Kim, J. S. (2014). Enhancement of alcohol metabolism by sprouted peanut extract in SD rats. Preventive Nutrition and Food Science, 19, 1–4. 24772402 10.3746/pnf.2014.19.1.001 PMC3999802

[fsn33520-bib-0077] Shah, M. , Paulson, D. , & Nguyen, V. (2018). Alcohol use and frailty risk among older adults over 12 years: The health and retirement study. Clinical Gerontologist, 41, 315–325. 28990855 10.1080/07317115.2017.1364681

[fsn33520-bib-0078] Sharma, S. , Bajgai, J. , Fadriquela, A. , Rahman, M. H. , Thuy, T. T. , Goh, S. H. , Kim, C. S. , Yu, K. , & Lee, K. J. (2020). The effect of a granule‐type anti‐hangover compound, quechung, on acute alcohol‐induced hangover in healthy subjects: A randomized crossover study. Korean Journal of Waters, 8(1), 22–33.

[fsn33520-bib-0079] Shati, A. A. , & Elsaid, F. G. (2009). Effects of water extracts of thyme (*Thymus vulgaris*) and ginger (*Zingiber officinale* roscoe) on alcohol abuse. Food and Chemical Toxicology, 47(8), 1945–1949. 19457445 10.1016/j.fct.2009.05.007

[fsn33520-bib-0080] Sheremeta, L. , & Haynuk, M. (2018). The apple pectin influence on biochemical and hematological parameters in animals with chronic alcohol intoxication. Reports of Vinnytsia National Medical University, 22, 280–284.

[fsn33520-bib-0081] Sierra‐Garcia, G. D. , Castro‐Rios, R. , Gonzalez‐Horta, A. , Lara‐Arias, J. , & Chavez‐Montes, A. (2014). Acemannan, an extracted polysaccharide from Aloe vera: A literature review. Natural Product Communications, 9(8), 1217–1221. 25233608

[fsn33520-bib-0082] Smith, H. S. , Wymer, J. P. , & Sang, C. N. (2009). Glutamate receptor antagonist. In H. S. Smith (Ed.), Current therapy in pain (1st ed., pp. 480–488). Elsevier Health Sciences.

[fsn33520-bib-0083] Srinivasan, S. , Dubey, K. K. , & Singhal, R. S. (2019). Influence of food commodities on hangover based on alcohol dehydrogenase and aldehyde dehydrogenase activities. Current Research in Food Science, 1, 8–16. 32914100 10.1016/j.crfs.2019.09.001 PMC7473379

[fsn33520-bib-0084] Subramanya, S. B. , Subramanian, V. S. , & Said, H. M. (2010). Chronic alcohol consumption and intestinal thiamin absorption: Effects on physiological and molecular parameters of the uptake process. American Journal of Physiology‐Gastrointestinal and Liver Physiology, 299, 23–31. 10.1152/ajpgi.00132.2010 PMC2904112 20448146

[fsn33520-bib-0085] Thomson, A. D. , Heap, L. C. , & Ward, R. J. (2017). Alcohol‐induced malabsorption in the gastrointestinal tract. In Alcohol and the gastrointestinal tract (pp. 203–218). CRC Press.

[fsn33520-bib-0086] Uto‐Kondo, H. , Hase, A. , Yamaguchi, Y. , Sakurai, A. , Akao, M. , Saito, T. , & Kumagai, H. (2018). S‐allyl‐L‐cysteine sulfoxide, a garlic odor precursor, suppresses elevation in blood ethanol concentration by accelerating ethanol metabolism and preventing ethanol absorption from gut. Bioscience, Biotechnology, and Biochemistry, 82, 724–731. 29616890 10.1080/09168451.2018.1447357

[fsn33520-bib-0087] Uto‐Kondo, H. , Sakurai, A. , Ogawa, K. , Yamaguchi, Y. , Saito, T. , & Kumagai, H. (2020). Suppressive effect of shiitake extract on plasma ethanol elevation. Nutrients, 12, 2647. 32878044 10.3390/nu12092647 PMC7551921

[fsn33520-bib-0088] Verster, J. C. , Stephens, R. , Penning, R. , Rohsenow, D. , Mc Geary, J. , Levy, D. , Mc Kinney, A. , Finnigan, F. , Piasecki, M. T. , & Adan, A. (2010). The alcohol hangover research group consensus statement on best practice in alcohol hangover research. Current Drug Abuse Reviews, 3, 116–126. 20712593 10.2174/1874473711003020116 PMC3827719

[fsn33520-bib-0089] Videla, L. A. , & Guerri, C. (2017). Glutathione and alcohol. In J. Vina (Ed.), Glutathione: Metabolism and physiological functions (pp. 57–68). CRC Press.

[fsn33520-bib-0090] Wang, F. , Li, Y. , Zhang, Y. J. , Zhou, Y. , Li, S. , & Li, H. B. (2016). Natural products for the prevention and treatment of hangover and alcohol use disorder. Molecules, 21(1), 64. 26751438 10.3390/molecules21010064 PMC6274469

[fsn33520-bib-0091] Warner, D. R. , Liu, H. , Miller, M. E. , Ramsden, C. E. , Gao, B. , Feldstein, A. E. , Schuster, S. , Mcclain, C. J. , & Kirpich, I. A. (2017). Dietary linoleic acid and its oxidized metabolites exacerbate liver injury caused by ethanol via induction of hepatic proinflammatory response in mice. The American Journal of Pathology, 187, 2232–2245. 28923202 10.1016/j.ajpath.2017.06.008 PMC5808136

[fsn33520-bib-0092] Wilson, D. F. , & Matschinsky, F. M. (2020). Ethanol metabolism: The good, the bad, and the ugly. Medical Hypotheses, 140, 109638. 32113062 10.1016/j.mehy.2020.109638

[fsn33520-bib-0093] Wilson, M. , & Braillon, A. (2018). Does moderate alcohol consumption really have health benefits? BMJ, 362, K3888. 30224550 10.1136/bmj.k3888

[fsn33520-bib-0094] World Health Organization Global status report on alcohol and health 2018‐Executive summary. file:///C:/Users/LAB5/Downloads/WHO‐MSD‐MSB‐18.2‐eng.pdf

[fsn33520-bib-0095] Yan, S. L. , Yang, H. T. , Lee, H. L. , & Yin, M. C. (2014). Protective effects of maslinic acid against alcohol‐induced acute liver injury in mice. Food and Chemical Toxicology, 74, 149–155. 25301236 10.1016/j.fct.2014.09.018

[fsn33520-bib-0096] Yoon, T. J. , Jo, S. Y. , Lee, S. J. , Kim, E. Y. , Shin, K. S. , & Suh, H. J. (2011). Effect of herbal composition, DTS20 on alcohol degradation and anti‐inflammatory activity. KSBB Journal, 26, 433–438.

[fsn33520-bib-0097] You, Y. , Lee, H. , Chung, C. , Lee, M. J. , & Jun, W. (2016). Effect of mixture including hot water extract of Houttuynia cordata Thunb on ethanol‐induced hangover in rats. Journal of the Korean Society of Food Science and Nutrition, 45, 1508–1512.

[fsn33520-bib-0098] Zhou, C. , Li, J. , Mao, K. , Gao, J. , Li, X. , Zhi, T. , & Sang, Y. (2019). Anti‐hangover and anti‐hypertensive effects in vitro of fermented persimmon juice. CyTA‐Journal of Food, 17, 960–966.

[fsn33520-bib-0099] Zhu, R. , Hou, Y. , Sun, Y. , Li, T. , Fan, J. , Chen, G. , & Wei, J. (2017). Pectin penta‐oligogalacturonide suppresses intestinal bile acids absorption and downregulates the FXR‐FGF15 axis in high‐cholesterol fed mice. Lipids, 52, 489–498. 28474246 10.1007/s11745-017-4258-x

[fsn33520-bib-0100] Zhu, R. G. , Sun, Y. D. , Hou, Y. T. , Fan, J. G. , Chen, G. , & Li, T. P. (2017). Pectin penta‐oligogalacturonide reduces cholesterol accumulation by promoting bile acid biosynthesis and excretion in high‐cholesterol‐fed mice. Chemico‐Biological Interactions, 272, 153–159. 28549616 10.1016/j.cbi.2017.05.018

[fsn33520-bib-0101] Zhu, Z. , Shi, Z. , Xie, C. , Gong, W. , Hu, Z. , & Peng, Y. (2019). A novel mechanism of gamma‐aminobutyric acid (GABA) protecting human umbilical vein endothelial cells (HUVECs) against H2O2‐induced oxidative injury. Comparative Biochemistry and Physiology Part C: Toxicology & Pharmacology, 217, 68–75. 30500452 10.1016/j.cbpc.2018.11.018

